# Engineering the interlayer exchange coupling in magnetic trilayers

**DOI:** 10.1038/srep16844

**Published:** 2015-11-24

**Authors:** Ching-Hao Chang, Kun-Peng Dou, Ying-Chin Chen, Tzay-Ming Hong, Chao-Cheng Kaun

**Affiliations:** 1 Research Center for Applied Sciences, Academia Sinica, Taipei 11529, Taiwan; 2 Department of Physics, National Tsing Hua University, Hsinchu 30013, Taiwan

## Abstract

When the thickness of metal film approaches the nanoscale, itinerant carriers resonate between its boundaries and form quantum well states (QWSs), which are crucial to account for the film’s electrical, transport and magnetic properties. Besides the classic origin of particle-in-a-box, the QWSs are also susceptible to the crystal structures that affect the quantum resonance. Here we investigate the QWSs and the magnetic interlayer exchange coupling (IEC) in the Fe/Ag/Fe (001) trilayer from first-principles calculations. We find that the carriers at the Brillouin-zone center (belly) and edge (neck) separately form electron- and hole-like QWSs that give rise to an oscillatory feature for the IEC as a function of the Ag-layer thickness with long and short periods. Since the QWS formation sensitively depends on boundary conditions, one can switch between these two IEC periods by changing the Fe-layer thickness. These features, which also occur in the magnetic trilayers with other noble-metal spacers, open a new degree of freedom to engineer the IEC in magnetoresistance devices.

The ability to tune the magnitude and sign of IEC^1^,^2^,^3^,^4^,^5^,^6^,^7^ in the giant magnetoresistance (GMR) system^8^,^9^,^10^,^11^, where ferromagnetic side layers (SL) are separated by a normal metal (NM) spacer, is key for developing nano-magnetoresistance devices. Reducing the device size can enhance the recording density, but it is often plagued by a dramatic increase in recording noise due to this coupling^12^,^13^. The carriers in the NM spacer, confined spin-dependently by both SLs, form the quantum well states (QWSs) and mediate the IEC. Via varying the NM thickness, the energy levels of QWSs can be shifted to cross the Fermi level, leading to the oscillatory behavior of the IEC^14^,^15^. It is well known that the carriers located at the belly of the NM Fermi surface induce the long-period oscillation (~10–18 Å or 5–9 monolayers (MLs))^3^,^16^ from the QWSs crossing. However, the carriers at the neck of the NM Fermi surface are related to the short-period oscillation (~4–6 Å or 2–3 MLs)^16^,^17^,^18^,^19^, but the microscopic mechanism is not clear up to now.

The predefinition of the SL nature can change the IEC significantly^20^,^21^,^22^,^23^,^24^. For example, by MgO capping, a proposed IEC period of 3 Cr MLs in the Fe/Cr/Fe trilayer^25^ is indeed observed^26^, due to the modification of conditions for forming the stationary waves in Cr^26^,^27^. Altering the SL thickness thus can offer an efficient way to tailor the phases at the interfaces and the IEC. An *ab-initio* study has indicated that the SL-thickness change can redistribute the weight between the different period oscillations in the IEC^19^. However, detailed understanding from first-principle calculations and the QWSs picture is still limited.

In this work, we investigate the relation between IEC and QWSs in the Fe/Ag/Fe sandwiches by using first-principles density-functional calculations. We firstly demonstrate that the NM QWSs at the neck of the NM Fermi surface are hole-like, which are qualitatively different to the electron-like QWSs located at the belly. The contributions of different classes QWSs in the IEC depend sensitively on the boundary conditions, hence we predict that the IEC oscillation can be changed dramatically and exhibits period-switching behavior as the SL thickness is altered.

## Results and Discussion

### Band structures, density of states, and magnetic coupling

**Figure 1:**
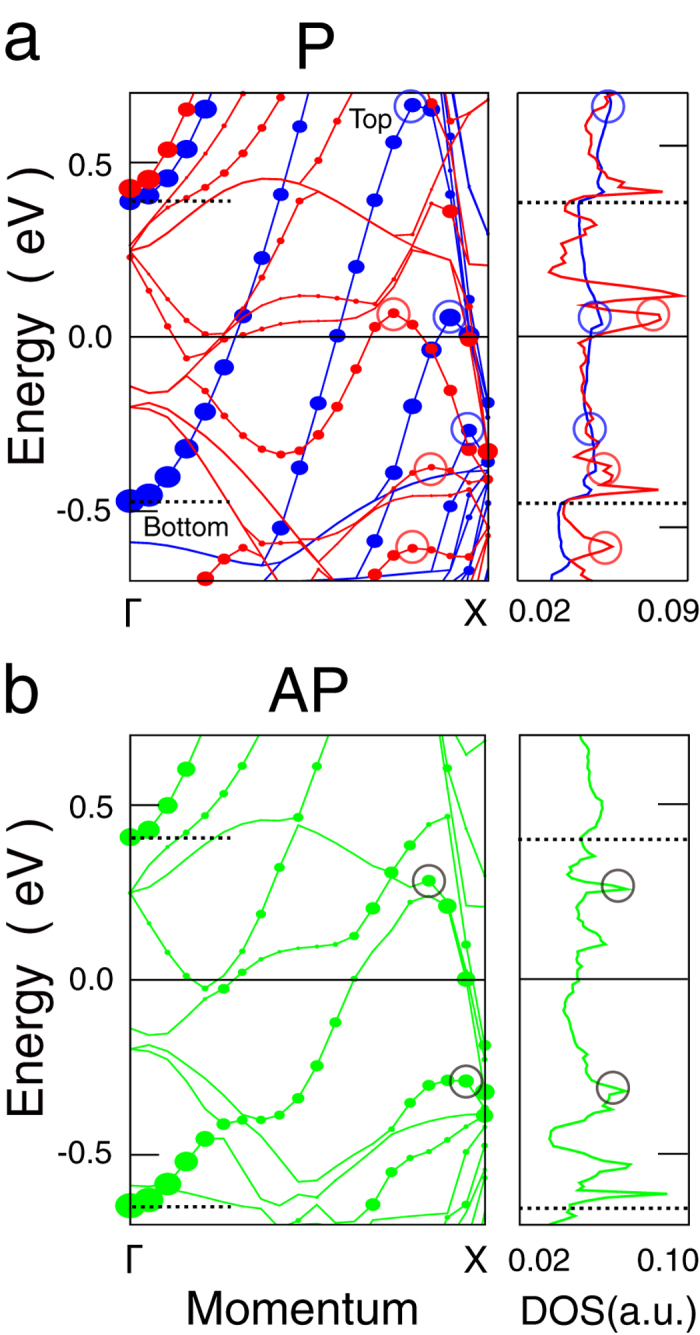
The band structures and the density of states (DOSs) of the Fe_3_/Ag_6_/Fe_3_ trilayer. The band structures (left) and the DOSs on the *p*_*z*_-orbital (right) of the system in (**a**) P and (**b**) AP magnetic configurations, respectively. The weightings of the *p*_*z*_ orbital of the majority (blue dots), the minority (red dots), and the AP states (green dots) in the Ag spacer are projected on the band dispersions.

To demonstrate the connection between quantum resonances and the IEC, we take the Fe_3_/Ag_6_/Fe_3_ trilayer as an example (see Methods). Figure 1 shows the band structures and density of states (DOSs) on the *p*_*z*_-orbital of the system in parallel-alignment (P) and anti-parallel-alignment (AP) configurations. In the P configuration, there are minority-spin and majority-spin carriers, which are antiparallel and parallel to the magnetization of Fe layers, respectively. The minority meets the band gap of the Fe layer and is trapped between the Fe-Ag interfaces to form the QWS, while the majority can hybridize with Fe states and forms the QWS between the boundaries of the trilayer. In the AP configuration, however, two spin carriers with opposite spin directions form the QWSs with degenerate energies since they undergo the same but spatially inverted confinement potentials.

The weightings of *p*_*z*_ orbital of the majority (blue dots), the minority (red dots), and the AP states (green dots) in the Ag spacer are projected on the band dispersions. The band without weighting (located around 0.3 eV) is the Fe-minority surface states (SSs) with *d*_*xz*_ and *d*_*yz*_ characters. Since the symmetry in such SSs is different from that of the Ag states, they hardly penetrate into the Ag layer to mediate IEC. The bands with a weak weighting (sat at around 0.12 and 0.1 eV) are the Fe-minority SSs with 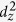 character. Although they share the same Δ_1_ symmetry as the Ag states and can enter the Ag spacer^28^, they hybridize weakly and sit above the Fermi level in our thin Fe layer system so that their contribution to IEC can be neglected.

The parabolic band dispersions thus indicate the appearances of these QWSs^29^, displayed in the left panels of Fig. 1. When the parabola band points upward (downward), that is the band bottom (top), denoted by a dashed line (a circle), the effective mass of the carriers is positive (negative), forming the electron-like (hole-like) QWS at Γ (the edge of Brillouin zone). These extremes correspond to peaks and steps of the DOSs on the *p*_*z*_-orbital, as shown in the right panels of Fig. 1. Therefore, around the Fermi level, the P configuration of the system has higher DOSs (the band tops occur) than the AP configuration.

The IEC constant *J* per area A can be determined by the difference in energy between these configurations


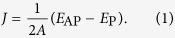


When *J* is positive (negative), the coupling is ferromagnetic (antiferromagnetic) and the SL magnetizations favor P (AP) configuration. The constant *J* is affected by the DOS difference at the Fermi level because of 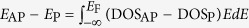
5,6. As the QWSs cross the Fermi level in P, they boost DOS_*P*_(*E*_F_) and lead to *J* < 0, and the Fe_3_/Ag_6_/Fe_3_ trilayer selects the AP configuration to lower the total energy.

### Hole-like quantum well states

For the IEC in the magnetic trilayer with a noble-metal spacer, pioneering works have identified that the NM QWSs, located at Γ (the Fermi-surface belly), lead to the long-period (~5–9 MLs) oscillation and are electron-like^3^,^16^. The NM QWSs, close to the zone edge (the Fermi-surface neck), are also found to generate the short-period (~2–3 MLs) oscillation^16^,^17^, but the microscopic understanding is absent. Our results in Fig. 1 suggest their existence and indicate that they are hole-like.

**Figure 2:**
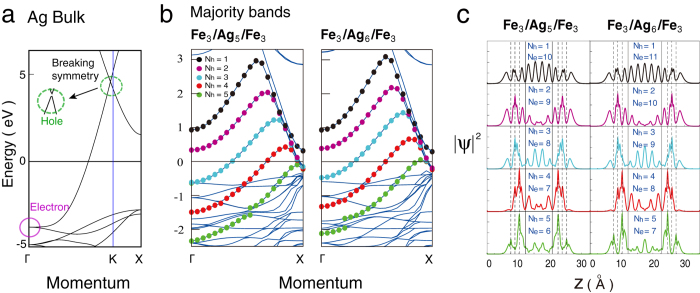
Formation of the hole-like QWSs in the GMR system. (**a**) The band structure of bulk Ag. The electron-like and hole-like carriers in the band structure are indicated by the solid and dashed circles, respectively. (**b**) The majority states of Fe_3_/Ag_5_/Fe_3_ and Fe_3_/Ag_6_/Fe_3_ trilayers in the P configuration. The N_h_ denotes the quantum number of hole-like QWS. (**c**) Plane-averaged charge densities of representative majority QWSs denoted in panel (**b**). The Fe layers (interfacial Ag layers) are indicated by dotted (solid) vertical lines.

To compare the different origins of hole- to electron-like QWSs, the band structure of Ag bulk and the majority bands in the P configuration of Fe_3_/Ag_6_/Fe_3_ trilayer are shown in Fig. 2a,b, respectively. For the band bottom at Γ referring to the electron-like state, the quantum confinement raises its energy as 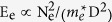 where N_e_ denotes quantum number, 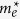 is the effective mass, and D is the confinement width^30^. Then the bottom of QWS bands can cross the *E*_F_ in the suitable width D resulting in the drastic modulation in DOSs.

The hole-like states, located near the band top, can also be produced by the intersection near the symmetry point **K**, since the trilayer system breaks the Ag lattice symmetry and transforms it into a pair of band bottom and top^31^,^32^ (see the dashed circle in Fig. 2a). As we introduce the quantum confinement, the energy of the top which refers to the hole-like state reduces as 
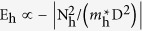
 and can cross the *E*_F_ to influence the DOSs. An example is shown in Fig. 2b, the QWS with N_h_ = 5 evolves from below to above the *E*_F_ when increasing the Ag spacer from 5 to 6 MLs.

Although the electron-like and hole-like QWSs locate at different regions in the k space, they belong to the same Ag band located at its bottom and top, respectively (see Fig. 2b). Their quantum numbers N_e_ and N_h_ tune the Ag states, and result in the mixing of two kinds of oscillation in the Ag-band spatial density profiles (see Fig. 2c). The N_h_ and N_e_ are responsible for the peaks of envelope and rapid oscillations, respectively, as N_h_ < N_e_ for the QWSs shown in Fig. 2c. The envelope oscillation that modifies the wavefunction of electron-like QWS has been detected by photoemission on noble-metal slabs^33^,^34^, but its origin is debated^15^,^35^. Here we attribute it to the wavefunction of hole-like QWS.

### Tailoring the magnetic coupling

**Figure 3:**
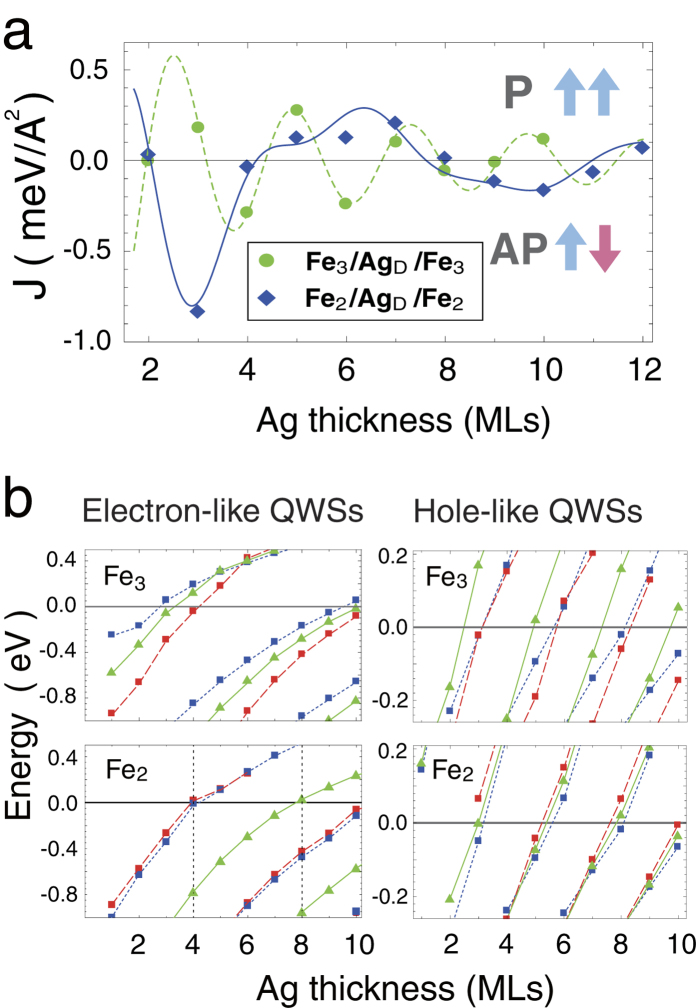
The IEC period switches due to SL-thickness modulation that shifts the QWSs spectra. (**a**) The bilinear coupling constant *J* as a function of Ag thickness. The solid and dashed lines are the fittings from equation (2). (**b**) Energies of the electron-like and the hole-like QWSs as functions of Ag thickness D in the Fe_3_/Ag_D_/Fe_3_ and the Fe_2_/Ag_D_/Fe_2_ trilayers. The blue (red) squares denote the majority (minority) QWSs in the P configuration and the green triangles represent the QWSs in the AP configuration. The lines in (**b**) connect the QWSs within the same N_h_ (the right panels) and N_e_ = *D* − *M* with M an integer (the left panels).

Since the QWS formations are sensitive to the boundary conditions, this characteristic provides an efficient way to tune the IEC by altering the SL thickness. Figure 3a shows the bilinear coupling constant *J* as a function of Ag thickness, which is calculated from first-principles (see Methods) according to equation (1). These data are consistent with the measured IEC periods^6^,^36^,^37^ and the predictions from analyzing the Fermi-surface topology^3^. They can be fitted by the following formula^26^,^27^,


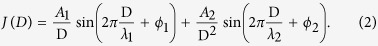


The first (second) term of equation (2) is the dominant (moderate) oscillation with the period *λ*_1_ (*λ*_2_), the phase *ϕ*_1_ (*ϕ*_2_), the amplitude *A*_1_ (*A*_2_), and a slower (faster) decay rate D^−1^ (D^−2^). The decay rate D^−2^ was obtained in a simplified case where the QWSs that mediate the IEC only exist in the P configuration^4^, while the D^−1^ rate comes from the inclusion of QWSs that appear in the AP and enhance the IEC^27^. For Fe_3_/Ag_D_/Fe_3_ sandwiches, the dominant (moderate) IEC period is *λ*_1_ = 2.37 MLs (*λ*_2_ = 6.5 MLs), and the parameters are *A*_1_ = 1.42 (*A*_2_ = 0.2) and *ϕ*_1_ = 0.33*π* (*ϕ*_2_ = 0). As reducing the system to Fe_2_/Ag_D_/Fe_2_ trilayers, the periods switch to *λ*_1_ = 6.5 MLs and *λ*_2_ = 2.37 MLs, and the parameters change to *A*_1_ = 1.57, *A*_2_ = 2.35, *ϕ*_1_ = 0.6*π*, and *ϕ*_2_ = 0.93*π*.

To understand these features from the QWSs, we plot the calculated energies of the electron- and hole-like QWSs, respectively from the band bottoms and tops of Fig. 1, for Fe_3_/Ag_D_/Fe_3_ and Fe_2_/Ag_D_/Fe_2_ sandwiches in Fig. 3b, where the blue (red) squares denote the majority (minority) QWSs in the P configuration and the green triangles represent the QWSs in the AP configuration. The electron-like (hole-like) QWS remarkably enhances the system energy and impacts the IEC when it approaches from below (is near) *E*_F_ inducing steps (peaks) in DOSs.

For the Fe_3_/Ag_D_/Fe_3_ trilayers, the electron-like QWS, triangles (squares) in the up-left panel of Fig. 3b, passes *E*_F_ at D = 3 (4) and 10 (9), resulting in the ferromagnetic (antiferromagnetic) couplings in Fig. 3a. As the passing period is 6.5 MLs, for D = 5 to 8 no electron-like QWS appears near *E*_F_, where the hole-like QWSs around *E*_F_ dominate the IEC. The hole-like QWSs, triangles (squares) in the up-right panel of Fig. 3b, approaches *E*_*F*_ at D = 5 (6) and 7 (8), leading to the ferromagnetic (antiferromagnetic) couplings in Fig. 3a, with a period of 2.4 MLs.

The Fe_2_/Ag_D_/Fe_2_ system exhibits a different IEC oscillation, for the SL modulation changes the formation conditions of the QWSs. The hole-like QWSs, shown in the down-right panel of Fig. 3b, come near *E*_F_ at the same Ag thicknesses, weakening their contribution to the IEC. The electron-like QWSs, a triangle (squares) shown in the down-left panel of Fig. 3b, cross *E*_F_ at D = 8 (4 and 10), causing the sign change of the IEC as plotted in Fig. 3a, with a period of 6.5 MLs. Therefore, two species of QWSs appearing at *E*_F_ with periods of 2.4 and 6.5 MLs (Fig. 3b) result in a double-period oscillation in IEC (Fig. 3a), where the well separated AP and P QWSs provide the period of *λ*_1_.

## Summary

We have confirmed the hole-like QWSs, stemming from the lattice-symmetry breaking and the layer confinement, as the origin of the short-period oscillation in the IEC of Fe/Ag/Fe sandwich. Modulating the Fe-layer thickness, the shifts of the energies of the hole-like QWSs and electron-like QWSs result in a dramatic change of the IEC, causing the period-switching behavior. This trend can be extended to the GMR system with other noble-metal spacers, such as Au and Cu (see Supplementary Figure S2), since they share the same face-centered cubic structure and can generate the same QWSs. The magnetic properties of these systems are gathering attentions for their potentials in spintronics^38^,^39^,^40^,^41^. Our results provide an efficient way to tailor their magnetic coupling.

## Methods

The unit cell consisted of the tetragonal bcc-like sandwich, where two or three Fe MLs formed each side layer and a Ag spacer ranging from 1 ML to 12 MLs was stacked in between (see Supplementary Figure S1). The unit cells were separated by at least 15 Å of vacuum and repeated periodically, and Fe atoms in the Fe SL were assumed to be ferromagnetic ordered. The in-plane lattice constant was fixed at 2.89 Å. We optimized the supercells of Fe_2_/Ag_D_/Fe_2_ and Fe_3_/Ag_D_/Fe_3_, with D = 1, 2, 3, 4 by using the Vienna *ab initio* simulation package (VASP), within the generalized gradient approximation. The optimization was reached when the residual force was less than 10^−2^ eV/Å. The lattice structures for D > 4 were constructed based on the structure of D = 4, with D − 4 additional Ag MLs inserted at the spacer center with an equal atomic distance of 2.13 Å. To calculate the total energies, the Brillouin zone summation was performed with a 32 × 32 × 1 k-point grid, the plane-wave energy cutoff was 400 eV, and the convergence criteria was less than 10^−3^ meV. The sufficiently large number of k points and high accuracy of total energy calculation were needed to ensure that the estimated error in IEC was less than 10^−1^ meV^42^,^43^.

## Additional Information

**How to cite this article**: Chang, C.-H. *et al.* Engineering the interlayer exchange coupling in magnetic trilayers. *Sci. Rep.* **5**, 16844; doi: 10.1038/srep16844 (2015).

## Supplementary Material

Supplementary Information
