# Supplementary material for: Engineering the interlayer exchange coupling in magnetic trilayers
Source: Sci Rep. 2015 Nov 24;5:16844. doi: 10.1038/srep16844 (PMC4657024; doi:10.1038/srep16844)
Supplement: Supplementary Information [file srep16844-s1.pdf]

Engineering the interlayer exchange coupling in magnetic trilayers

Ching-Hao Chang, Kun-Peng Dou, Ying-Chin Chen, Tzay-Ming Hong, and Chao-Cheng Kaun

Supplementary Information

S1. Unit cell geometry

The lattice structure of Fe is body centered cubic (bcc) while of Ag is face centered cubic (fcc). Therefore, the Ag (001) rotates in x-y plane of an angle $\pi/4$ to join two Fe layers (see Fig.S1). Both sides of trilayer are exposed to vacuum.

S2. Magnetic sandwiches with different noble-metal spacers

To demonstrate that our results, namely (1) the appearance of hole-like QWSs and (2) the period-switching behavior in IEC, are general in magnetic trilayers with noble-metal spacers, the similar calculations of the band structures and density of states (DOSs) are performed for Fe/Au/Fe (001) and Co/Cu/Co(001) systems. The results are shown in Fig.S2.

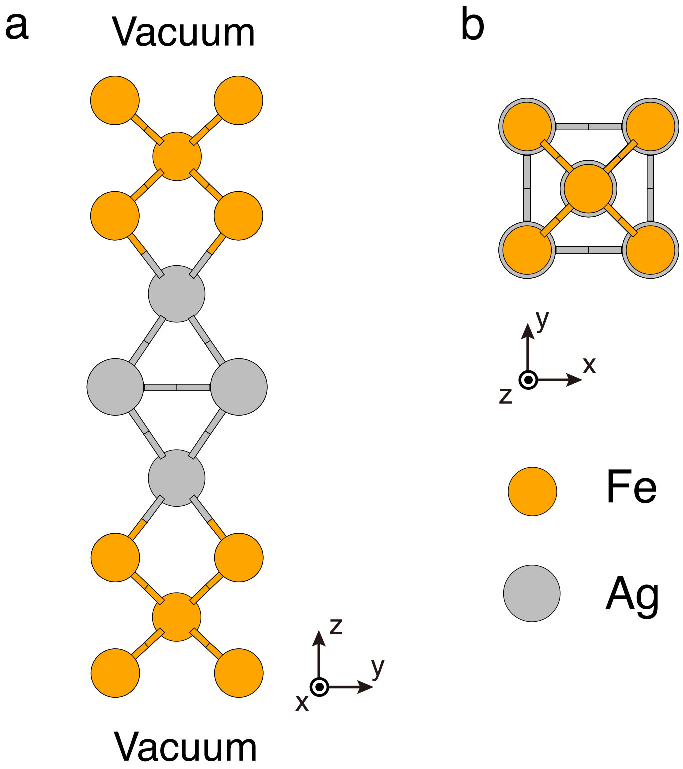

Figure S1: **Unit cell used for $Fe_3/Ag_3/Fe_3$ trilayer a**, Longitudinal direction. **b**, Cross section.

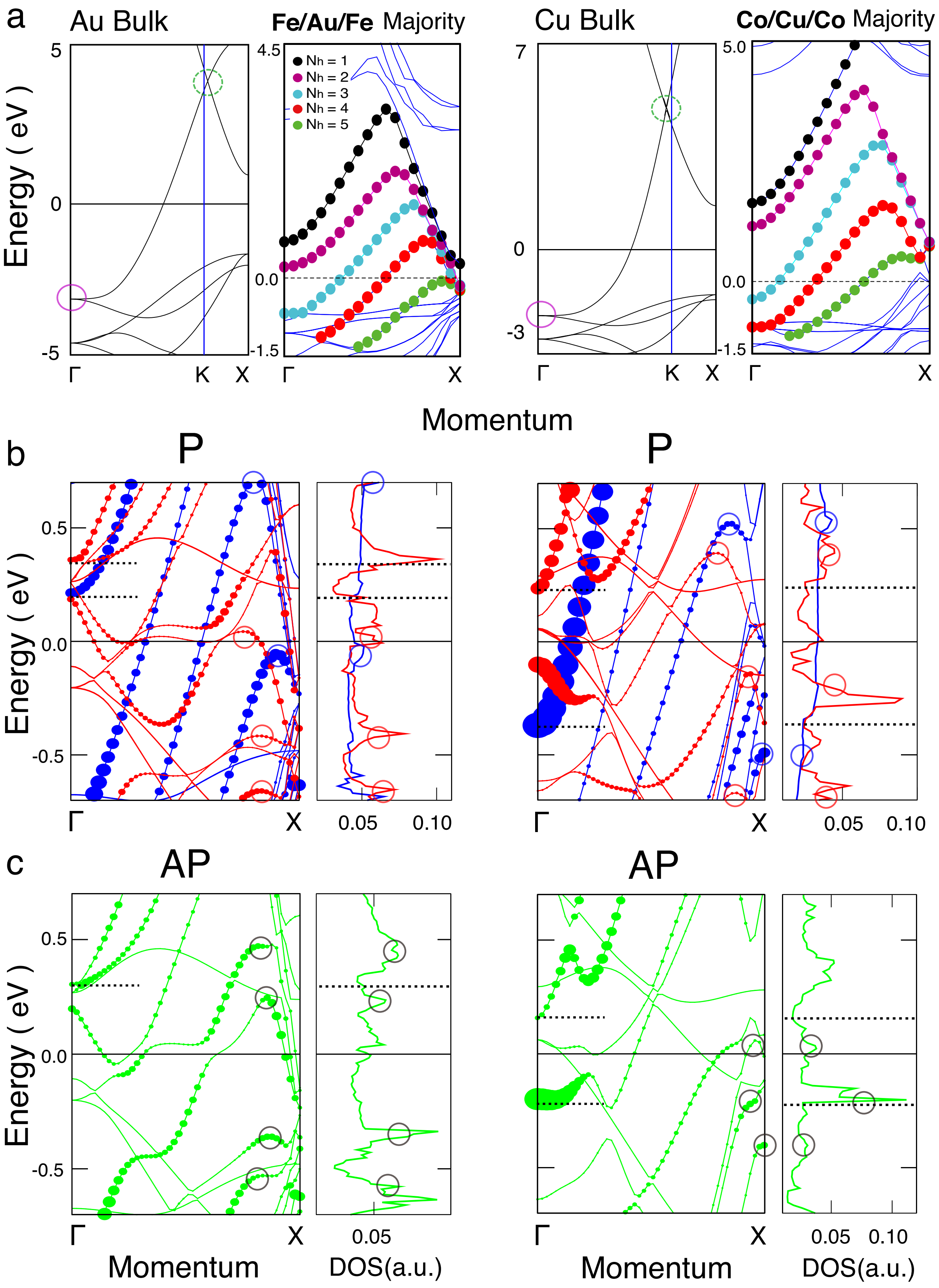

Figure S2: **Different magnetic trilayers with noble-metal spacers a**, Band structure of Au and Cu bulks, and the spectra for majority states in $Fe_3/Au_6/Fe_3$ and $Co_3/Cu_3/Co_3$ trilayers. **b, c**, The band structures and DOSs of both trilayers with P and AP configurations.
